# Efficiency of different airborne disinfection products for reducing the risk of *Aspergillus fumigatus* infection from hospital false ceiling exposure

**DOI:** 10.1186/2047-2994-4-S1-P50

**Published:** 2015-06-16

**Authors:** S Loeffert, M-P Gustin, P Cassier, P Bouche, C Lion, M Massacrier, M Perraud, P Vanhems

**Affiliations:** 1 UMR CNRS 5558 Biometry and Evolutive Biology Laboratory, Claude Bernard 1 University, Lyon, France; 2 Institut of Pharmaceutical Sciences and Biology (ISPB), Claude Bernard 1 University, Lyon, France; 3 Environmental Safety and Biology Laboratory , Edouard Herriot Hospital, Lyon, France

## Introduction

In hospital, false ceilings are a source of *Aspergillus.* A huge amount of spores may be suspended specially during their demolition near immunocompromised patients who are at risk to develop severe invasive aspergillosis (IA). Airway disinfection allows the no directed spraying of a disinfectant on surfaces contained into a determined volume.

## Objectives

The aim of this study was to evaluate the antifungal efficacy of a new airborne disinfectant (AD) on hospital false ceilings compared to another AD usually used, before major deconstruction work.

## Methods

The antifungal activity of the two AD was assessed in 4 rooms of an hospital ward in a building intended to be demolished. The two AD tested were based on hydroxyacetic acid for AD#1 and on peracetic acid and H_2_O_2_ for AD#2. Each AD was randomly applied in rooms as recommended by the manufacturer with comparable trial conditions (location and contact time with AD). Fungal contamination (F*_c_*) of the top of false ceiling slabs was assessed using moistened swabs before and after application of the AD. The environmental contamination was then extracted and inoculated on YGC agar. On each plate *A. fumigatus* and *A.niger* were counted. Contamination rates (*C_R_*) were compared after logarithmic transformation by nonparametric tests.

## Results

A total of, 11 false ceiling tiles were sampled before and after AD in each room (a total of 88 tiles). Before disinfection, *C_R_* of rooms were not different. For AD#1 the *C_R_* for *A. fumigatus* was 1.86 before AD and 1.59 log_10_ after (*p*=0.80), for *A. niger the C_R_* was 0.45 before and 0.24 log_10_ after (*p* =0.24). For AD#2 the *C_R_* for *A. fumigatus* was of 1.69 before AD and 0.80 log_10_ after (*p*=0.01), for *A. niger* the *C_R_* was of 0.60 before AD and 0 log_10_ after (*p* =0.001). Concerning AD#1, the *C_R_* reduction was then 0.89 and 0.60 log_10_ for *A. fumigatus* and *A. niger* respectively.

## Conclusion

The new AD#2 was the most efficient to reduce F*_c_* of hospital false ceiling slabs before demolition. These results will be of major help to choose the best AD procedure for improving the control of *A. fumigatus* risk and ultimately prevent IA in our patients during construction works of our hospital.

## Disclosure of interest

S. Loeffert Grant/Research support from: ANIOS Laboratories. The authors thank ANIOS Laboratories for their participation in this study by providing free of charge products. Anios was not involved in the study design, analysis results or discussion. , M.-P. Gustin: None declared, P. Cassier: None declared, P. Bouche: None declared, C. Lion: None declared, M. Massacrier: None declared, M. Perraud: None declared, P. Vanhems: None declared.

